# Assessing the association between subjective evaluation of space qualities and physiological responses through cinematic environments’ emotion-eliciting stimuli

**DOI:** 10.3389/fpsyg.2022.1012758

**Published:** 2023-01-12

**Authors:** Hamidreza Sakhaei, Ning Gu, Mehdi Azizmohammad Looha

**Affiliations:** ^1^Architectural Design, Modeling, and Fabrication Lab, Department of Architecture, Tarbiat Modares University, Tehran, Iran; ^2^UniSA Creative, Australian Research Centre for Interactive and Virtual Environments, University of South Australia, Adelaide, SA, Australia; ^3^Department of Biostatistics, Faculty of Paramedical Sciences, Shahid Beheshti University of Medical Sciences, Tehran, Iran

**Keywords:** physiological responses, cognitive emotion, spatial perception, film stimuli, environmental appraisal

## Abstract

**Objectives:**

Human perception of the built environment affects emotional and physiological states. This research focused on the association between evaluating a space’s visual qualities and physiological responses by mediating film contents to indicate the association between physiological indicators and assessing the quality of space in the presence of environmental stimuli.

**Method:**

Data collection was conducted using a psychological questionnaire and physiological indicators of heart rate (HR), blood pressure (BP), skin resistance level (SRL), and body temperature (BT) during the film screening. The ANOVA was conducted to compare different variables in the three films alongside linear regression to analyze the impact of variables on space quality. Spearman correlation coefficient analyses were performed to find the association between variables.

**Results:**

The descriptive statistics showed significant changes in psychological and physiological variables in films. Associations between the NAQ factor and physiological changes in HR, SBP, and DBP factors were significant. The results derived from the simple and multiple linear regressions depicted the significant impact of physiological factors on HR and BP on perceiving the quality of space.

**Conclusion:**

It was concluded that physiological changes caused by emotional arousal could be strongly associated with psychological assessments. Stimuli-affected video contents illustrating architectural spaces could efficiently extract the impact of physiological states and human cognitive responses to the space quality. Physiological characteristics related to the space appraisal could help realize the human-environment interaction in a multi-layered approach to the built environment and spatial cognition.

## 1. Introduction and background

The built environment’s effects on the occupants are of great interest to architects (Hu and Roberts, 2020), mainly because of the critical values of architectural perception (both mental and sensory) embodied in biological nature (Kirk et al., 2009; Wiesmann and Ishai, 2011; Vartanian et al., 2013, 2015). Human responses to environmental agents are divided into behavioral, psychological, and physiological categories (Steimer, 2002). With external stimuli in the environment, behavioral (Levitis et al., 2009) and physiological changes may emerge as an autonomic bodily reaction to a stimulus to depict the occurrence of environmental conditions outside the optimal border (Elliott and Quintino, 2019). While emotional responses are assumed to be meticulously associated with behavioral and cognitive targets (Mehrabian and Russell, 1974; Inzlicht et al., 2015), psychological responses refer to the changes in emotional and mental characteristics of people when exposed to various environmental stimuli (Persiani et al., 2021). Indoor built environments can generate conditions where exposure to architectural elements (e.g., geometry, lighting, color, texture, etc.) can psychophysiologically affect individuals (St-Jean et al., 2022).

Studies on the multi-domain approaches have shown that visual and auditory domains work cooperatively in inducing human perception and behavior (Torresin et al., 2018; Schweiker et al., 2020). Studying the relationship between film narrative and embodied cognition has unveiled the close association between physiological states and the physical environment (Kiss, 2015). Also, human perception of space is informed by experiencing it in movement, which led to many scholars considering the architectural space, movement, and film integrated into their experiments (Bruno, 2002). The simulation of the architectural space through the mediation of video clips or virtual environments has become feasible (Hong and Jeon, 2013; Hong et al., 2019; Park et al., 2020), which can help convey and articulate emotions from the potential of films and cinematic context (Bruno, 2002). Accordingly, the notion of movement is the fundamental principle of creating emotional feelings in which drawing attention to the motion can consequently evoke an emotion (Bruno, 2002). Noting that a person’s attentiveness is drawn to a novel environmental confrontation (Sakhaei, 2020), generating movements in a cinematic context can activate a multisensory experience. This activation signifies that human perception involves visual and auditory stimuli to interact and modify the overall attentiveness (Spence et al., 2014; Schreuder et al., 2016; Ba et al., 2020). As a result, the mediation of film stimuli can then lead to the perception and exploration of observed visual information and reveals individuals’ preferences of spatial qualities (Kaplan and Kaplan, 1989).

Owing to the undeniable impact of the built environment’s quality and design on humans (Aminah, 2018), we tried to find the relationships between physiological changes and the appraisal of the space quality. We relied on Zawidzki’s research that proposed a new definition of human subjective evaluation (HSE) of space retrieved from normalized averaged values in self-assessment questionnaires (Zawidzki, 2016a). The twenty items of the HSE test present a novel approach of subjective evaluation based on geometrical characteristics of an architectural space plan to measure the subjective appraisal, in which the evaluation is mainly related to the spatial perception rather than its aesthetic properties. These items are further explained in the methods section of the paper.

We also focused on four physiological states of blood pressure (BP), heart rate (HR), skin resistance level (SRL), and body temperature (BT). While physiological reactions have proven their influential role while observing film contents (Rooney et al., 2012), there is a lack of significant research to identify a robust relationship between physiology and normalized space quality perception through film mediation. Hence, we hypothesized that changes in assessing the quality of a space could be meaningfully associated with physiological states in the presence of an environmental stimulus. Through screening the film spaces in two different situations of pre-event and post-event to the participants, we conducted a multivariate quantitative and a qualitative experiment to capture the impact of spatial stimuli on the association between space quality perception and physiological responses. As a result, we focused on participants’ attention to evoke their emotional states and activate their sensory-motor by implementing unexpected stimuli to change the quality of space understanding. Moreover, we tried to quantitatively explore which physiological parameters are associated with the psychological assessment of spatial qualities by comparing responses from the pre-and post-event situations.

### 1.1. Space, stimuli, and human behavior

The human perception of architecture and built environment affects the emotional and physiological states of their immediate surroundings originating from the impact of sensory stimuli in space (Homolja et al., 2020). This perception is not generated by a sole isolated sense (Li et al., 2021); Instead, various sensory organs notice the environment’s physical characteristics and incorporate the physical information with multimodal perception to shape the so-called bottom-up cognitive process (Kayser and Logothetis, 2007). This cognitive process is then proved to be coordinated by emotion and human behavior (Talsma et al., 2010), consequently forming subjective perceptual experience (Choi et al., 2018).

While sensory input from the environmental interaction has a decisive role in feelings and behaviors (Turley and Milliman, 2000), various environmental characteristics in space like material and color can generate sensory input, which contributes to particular feedback in the spectator (Biggers and Pryor, 1982; Franz, 2006). For instance, textures and surfaces, scale and distance of an entrance, the shifting view as a person moves along the path, steps and inclines, the peripheral field of vision, and the empathy with the space are various visuo-spatial features affecting emotional states toward experiencing architecture (Barrie, 1997). Additionally, the architectonics of space elements affect emotional states for assessing spatial characteristics (Gifford, 2007). For instance, scale, angle, and element type (Kent et al., 2021), as well as material type, light and color of the interior space (Banaei et al., 2020) have shown their direct impact on emotional behavior and visual properties. Previous studies also pointed out that architectural characteristics like spatial openness (Hedge, 1982; Sundstrom et al., 1982) or enclosed and open spaces can affect mental satisfaction. Theorists in architecture mention complexity and order to be decisive in evaluating architectural quality (Weber, 1995), and balance in architectural elements can elevate cognitive function (Bruer, 1997). Therefore, the appearance of any space-affective stimulus to change architectural qualities can potentially lead to emotional behavior and cognitive function in physical or mediated spaces (Soleymani et al., 2009).

The spectator’s responses to the surrounding built environment can be the outcome of either visual, auditory, somatosensory, or olfactory stimuli (St-Jean et al., 2022). However, most of the existing research has focused on visual stimuli owing to the cultural and social strength of the visionary dominance (Hutmacher, 2019) or, specifically, the conventional dominance of sight in the realm of architecture (Spence, 2020). This dominance could be because vision is proven to be more sensitive than other senses to interpret space distribution (Fisher, 1968) and perceive an object in the environment (Godfroy-Cooper et al., 2015). Audio-visual studies demonstrate that congruent or incongruent multisensory stimuli can affect the appraisal of the environment by amplifying emotion and significantly enhancing emotional, cognitive, and behavioral responses to the environment (Schreuder et al., 2016). Choosing an affect-based film content requires intensity measurements and the type of affect anticipated from the observer (Soleymani et al., 2009). A previous experiment divided the movie contents into the categories of calm, average, and exciting to sufficiently inspect emotional arousal (Xu et al., 2008). As a result, it would be essential to identify and control the complexity, illumination, movements, character numbers in the film, and camera angles to better understand the impact of stimuli and human behavioral analyses in mediated contents during subjective appraisals (Carvalho et al., 2012).

During exposure to the audio-visual stimuli in the environment, attention is a crucial condition to be studied (Persiani et al., 2021). Accordingly, the human brain constantly oscillates between deep focus, a condition in which the mind temporarily disengages from the real world during extreme concentration on a task (Buetti and Lleras, 2016), and surrounding consciousness, when the mind is distracted by spatial stimuli (Wais et al., 2010). As a result, this oscillation between the occurrence of several attentions can be interconnected to human behavior (Fiebelkorn et al., 2018) so that the improvement of a cognitive engagement or attention can lead to diminished sensitivity to the visual events (Buetti and Lleras, 2016) and also declined situation awareness (Pizzamiglio et al., 2017). Additionally, the Chatterjee model points out the close relationship between attention, affective-emotional output, and the decision-making process by defining the five phases; 1. processing of simple factors, 2. attention to noticeable properties, 3. attention modulation, 4. feed-back/feed-forward processes bonding the attentional and attributional circuits, and 5. interference of the emotional systems (Chatterjee, 2003), all contextualized in architectural research (Coburn et al., 2017).

### 1.2. Physiology, emotion, and spatial cognition

The unconscious effect of physical spaces on psychological procedures (Hochberg and Brunswik, 1954) works in a way that the characteristics of the space and affective potentials provoke perceptions and sensations that cause behaviors and responses (Russell and Snodgrass, 1987; Vogels, 2008). On the other hand, emotion plays a decisive role in subjective environmental perception (Sakhaei et al., 2022b), which encompasses different positive or negative affective states that represent motivational phenomena from neuro-physiological, experiential, or expressive factors in the environment (Dormann, 2003). Multiple studies have highlighted the architecture-emotion interactions in which effective spatial stimuli arouse emotions (Altman and Stodols, 1987; Cohen and Areni, 1991; Cacioppo et al., 2000; Radberg and Steffner, 2003; Cho and Kim, 2017). Architecture as a built environment directly impacts human emotion, perception, behavior, preference, and brain response (Khaleghimoghaddam et al., 2022). Individuals’ judgments about subjective features of the environment like calm, security, boredom, or fear designate the affective and emotional states of the observer (Khaleghimoghaddam et al., 2022). With the presence of spatial stimuli to provoke emotional perceptions (Spreckelmeyer et al., 2006), the arousal level may elevate and subsequently impact affective appraisal of the environment or object (Michon et al., 2005; Liu and Jang, 2009). The emotional changes that lead to human cognitive appraisals can appear in terms of evaluating objects, events, behavioral tendencies, and physiological responses (Scherer, 2001).

Several theories, such as the James-Lange theory (James, 1884), Facial Feedback theory (Lanzetta et al., 1976), Cannon-Bard theory (Cannon, 1927), and Schachter-Singer theory (Schachter and Singer, 1962) point out the close relationship between emotion-triggered external stimuli and autonomous physiological reactions. The design characteristics of a space can have a short-term physiological effect once processed by the human sensory system (St-Jean et al., 2022). These sensory features associated with sensation felt from the architectural space characteristics (e.g., color, light, sounds, or textures) can engage the arousal level of human perception (Lang, 1988; Nasar, 1997; Sanoff, 2016; Cho and Kim, 2017). The physiological responses from emotional arousal (Kreibig, 2010) help detect auditory and visual signals in the environment and lead to the activation of the autonomic nervous system (ANS), which include both sympathetic (SNS) and parasympathetic (PNS) nervous systems (Li et al., 2021). The SNS activates in response to stress, while the PNS maintains the balance after the occurrence of stress (Li et al., 2021). High-precision biometric sensors can help study the physiological effects of spatial stimuli (Nkurikiyeyezu et al., 2019) to extract information on a biological system (Bronzino, 2005) in biomedical applications (Fong et al., 2013). The activation of the ANS system may lead to multiple physiological indicators such as BP (Lee et al., 2009), HR (Ulrich, 1981), SCL (Yin et al., 2018), and BT (Yoshihara et al., 2016; Ding et al., 2018), that can be reliable to investigate environmental quality (Li et al., 2021). Consequently, as emotion is reflected in all modes of human communication, the emotional recognition system is accurate when the user’s situation is considered (Picard et al., 2001), particularly during exposure to an environmental stimulus.

### 1.3. Films, psychophysiology, and spatial evaluations

According to the fundamentals of environmental psychology, human-environment behavior and correlation are influenced by mental processes (Radberg and Steffner, 2003; Sternberg and Wilson, 2006; Gifford, 2007; Pakzad and Bozorg, 2014). The affective environmental qualities that determine emotion can help people respond to the space in an emotional evaluation process (Sakhaei et al., 2022a), which signifies the environmental capability to change human affection (Russell and Lanius, 1984). Cinematic techniques like camera movement can organize the process of metaphorical mapping in which the inferential logic of image schemas will be able to convey conceptual knowledge of screened contents (Coëgnarts, 2017). The cognitive science theories corroborate that movement is perceived through motion recognition in peripheral vision (Finlay, 1982). According to Bruno, films are perfect mediums to illustrate the movement in space and articulate emotion (Bruno, 2002). She mentions the close interrelation between sight and site, as well as motion and emotion, especially in architecture (Bruno, 2002), meaning that the architecture should be experienced with a duration or path of movements. Since perception is proven to be action-oriented and cinema holds the capacity for many actions in space (Gibson and Carmichael, 1966), the exploration of fundamental physiological responses can be facilitated (Schreuder et al., 2016). Cinematic contexts can affect emotion as they are a valuable archive of emotional pictures and psychological sub-stories bonded to our built environment (Bruno, 2002). They also allow coordinated illustration of continuous, contextualized, and vibrant sets of stimuli to-be-remembered (Furman et al., 2007). Hence, cinema can act as a comprehensive framework to study the everydayness of architecture for human-environment examinations (Penz, 2017).

As mentioned, films can induce movements and actions in space by representing architectural elements where the impacts of environmental effects on viewers vary in sensitivity (Dijkstra et al., 2008). Hence, movement, space, action, and objects are key fundamentals for studying the cinematic context in terms of interaction and intersubjectivity (Gallese and Guerra, 2012). By conducting a psychological approach, a practical foundation can be provided for appraising visual quality based on different levels of the individuals’ sensitivity (Daniel and Vining, 1983). The appraisal of visual quality is highly dependent on environmental stimulation as it directly relates to brain development (Perry, 2002), while multisensory cinematic experience can provoke wide-ranging affective responses (Kaltwasser et al., 2019). Besides, the visual field analysis is a beneficial tool in spatial evaluation techniques to effectively apply in building analyses (Dalton, 2007). For instance, a previous study captured the association between the geometric properties of a space and emotional experiences using the semantic differential scaling technique (Franz et al., 2005). Another study presented interactive film content based on participants’ expressed emotions and peripheral physiological signals to distinguish user preferences (Wu et al., 2008).

**Figure 1:**
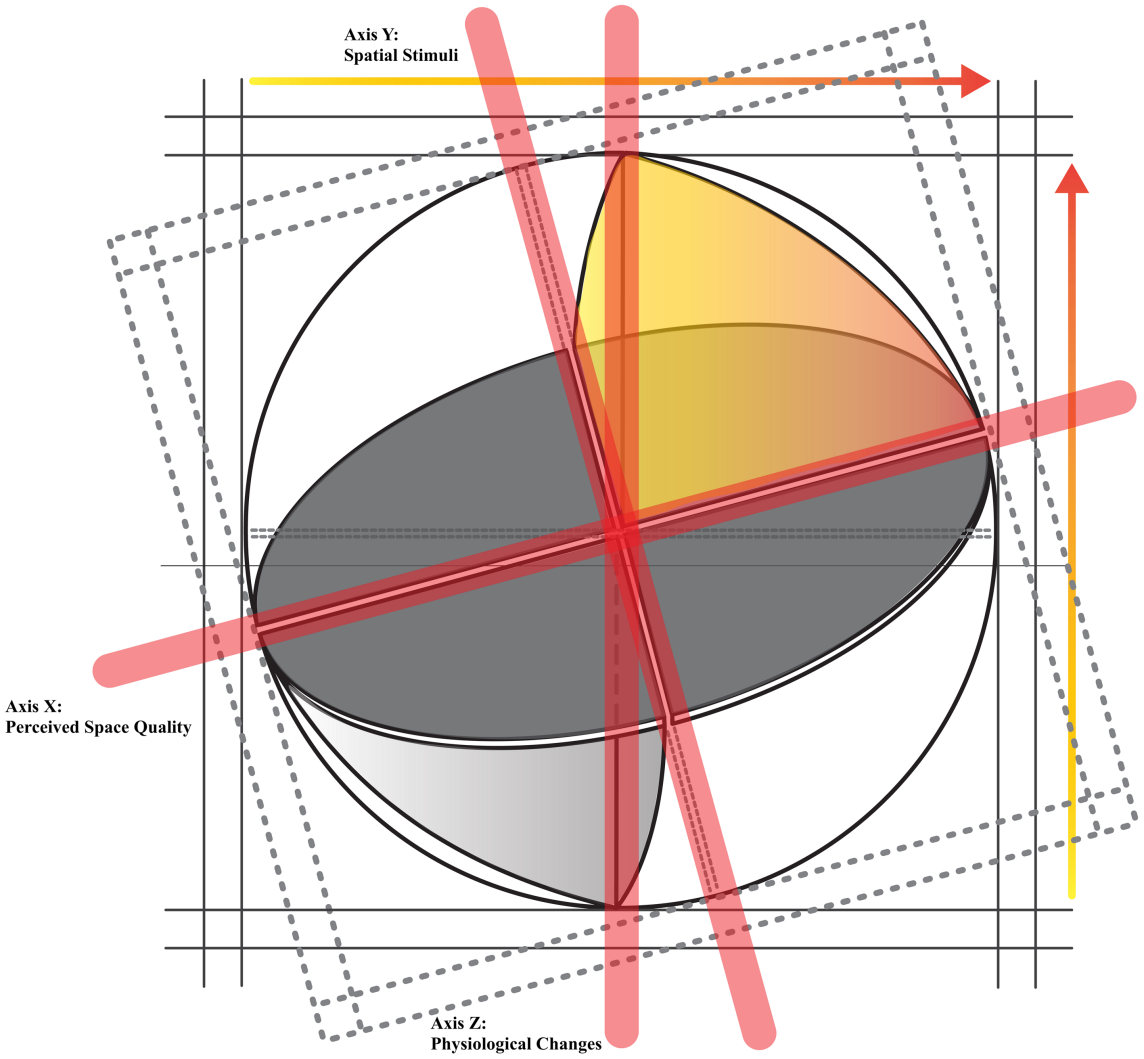
The present research’s theoretical model inspired by Mehrabian and Russell’s tripartite model of pleasure, arousal, and dominance (Bakker et al., 2014). The yellow–orange sector of the sphere illustrates the association between increased axes of Perceived Space Qualities, Spatial Stimuli, and Physiological Changes.

This research focused on finding the association between evaluating a space’s visual qualities and physiological responses by mediating film contents to simulate real physical spaces. A previous study (Zawidzki, 2016a,b) presented a new definition of subjective evaluation of a space founded on normalized averaged values. As mentioned in the literature, various studies tried to establish the connections between spatial cognition and psychophysiological responses. Nevertheless, very few current studies focused on various physiological feedback and subjective evaluation of the space’s qualities when impacted by environmental stimuli. In this regard, the normalized accumulated quality framework may highlight the physiological attributes of built environments when affected by unexpected events. Therefore, for the purpose of this research, we hypothesized that the four main physiological indicators of HR, SRL, BP, and BT can significantly correlate with assessing the quality of space in the presence of environmental stimuli. The remainder of the paper will try to inspect which physiological changes can affect people’s reactions to the visual characteristics of a space. In achieving this aim, we theorized in a model (Figure 1) that physiological changes can validate the role of spatial stimuli in improving the perception of the space’s qualities.

## 2. Methods and data collection

### 2.1. Study population and sampling method

We recruited 90 individuals of Iranian ethnicity for this trial (half female/male, mean age 26, SD 2.69, and range between 20 and 30 years old) who reported as students or employed. As discussed in the literature, the participants’ sensitivity to the observed built environment is highly reliant on the spatial exposure impact (Dijkstra et al., 2008), which led us to choose a homogenous age range to capture a more controlled behavioral and physiological feedback than adolescents or elderly subjects with potential differences in past experiences and affect assessments. We also selected an equal number of participants in gender to better control the homogeneity of psychophysiological responses. All the subjects reported that they had not watched the movies before the experiment to experience a novel sense of spatial perception. During the pre-test preparations, we briefed the entire assessment process to each participant. While we asked if they were under any medication, we aimed to ensure the process would be accomplished in a standard condition. Subjects reported they had sufficient sleep the night before the experiments and had a proper meal a few hours before the test. We also asked them to avoid smoking, drinking alcohol, or consuming caffeine-contained food or beverage at least 4 h before the test. The assessment protocol was validated by the Department of Physiology of the affiliated institution, and performed under the ethical standards as outlined in the 1964 Declaration of Helsinki and its later amendments or comparable ethical standards.

### 2.2. Editing the cinematic experiment

To identify standard film content in order to extract our desired physiological feedback, we asked different academics in the field of cinema and art studies. Based on the experts’ comments, we edited three films into shorter versions by considering several crucial criteria. First, we extracted specific scenes that represented a standard depiction of architectural spaces to properly gather viewers’ attention, meaning that the architectural characteristics would solely influence the audience by eliminating the effect of screenplay and dialogues. We called this criterion Space Narration, which refers to the content that gathers viewers’ attentiveness irrespective of the film’s script. Secondly, we implemented a disruptive and unexpected scene in the middle of each clip to create and before and after the situation; a pre-event scene with an everyday space screening the ordinary architectural spaces, and an event scene illustrating unexpected events that deconstruct the architectural elements depicted in the pre-event scene. We called this consideration Presence of Unexpected Events, which related to the previous criterion of Space Narration, owing to eliminating script impact to avoid storytelling and dialogues, would prioritize architectural space visualization and improve spatial cognitive load.

To fulfill the above two criteria, we relied on the formal form analysis system (Bordwell et al., 1993; Bordwell, 2013) to choose scenes with a continuous camera movement that captures one-point perspectives of spaces. Also, we chose the scenes in which the camera height and angle simulated an eye-level perspective to help participants feel like they were exploring the space themselves from point-of-view shots. The unexpected events in the middle of each film clip play the role of stimulus in the middle of pre-event and post-event scenes to bring emotional and physiological effects to the subjects. The Three films’ plot summaries and details about pre-event and event scenes are presented in Table 1.

**Table 1:** Films’ plot summaries and space-related characteristics.

Movie name	Plot summary	Pre-event scene details	Event scene details
Movie No. 1: *The Money Pit (1986)*	A young couple walk into a classic architecture house to see if it is worth buying. While they figure out the house needs redecoration to look new again, unexpected accidents happen during the redecoration process.	The young couple inspect the house’s different spaces to see if it satisfies their needs. After buying agreement, they begin redecorating the house to make it appropriate for living.	The front door collapses during the redecoration. The interior main stairs break down and electrical wiring in the kitchen catches fire, making the tiles crush. The bedroom chimney gets ruined when the husband tries to light the fire.
Movie No. 2: *Zathura: A Space Adventure (2005)*	A father and two kids live in a wooden house. When the kids’ father leaves the house after a business call, the lone kids confront peculiar events in the house after starting to play a weird board game.	A routine father-son play in the house portrays a happy weekend in a family. The kids run into the living room and kitchen, and climb the stairs as they feel like the house is a playground.	The house suddenly gets hit by several fireballs penetrating the rooftop, walls, and ceiling. To escape from fireballs, the frightened kids run away for shelter as they witness their house gets damaged.
Movie No. 3: *I, Robot (2004)*	The movie narrates the future in which robots work as human servants. Although there are rigorous guidelines to control their activities, a detective has to explore if a robot has violated the policies and murdered a person.	The detective walks into an empty house for the survey. He doubtfully moves through the corridor, climbs the stairs, and investigates a private room for evidence. At least for a while, he finds no strange activity.	An enormous robot destroys the walls and stairs and continues the demolishment until the entire house collapses. The detective runs away to save his life during the unexpected attack.

In order to control the film content in terms of sound and light, we chose two categories of scenes with different levels of sound and light attributed to pre-event versus post-event spaces. The pre-event scenes had a lower-intense sound level to induce a state of comfort and familiarization with the space atmosphere. Also, the light condition in pre-event scenes was noticeably dimmer than in the post-event scenes to illustrate the coziness and readability of space. On the contrary, the post-event scenes were chosen from scenes with a much-intensified level of sound and light to better represent the spatial stimuli and disruptive architectural elements. As mentioned, the selection of the film clips and the validity and feasibility of their final editions were guided by experts and academics in the fields of cinema and art studies. The films were revisited and finalized multiple times in order to properly convey the depiction of architectural qualities based on the pre-event and post-event scenes’ criteria.

**Figure 2:**
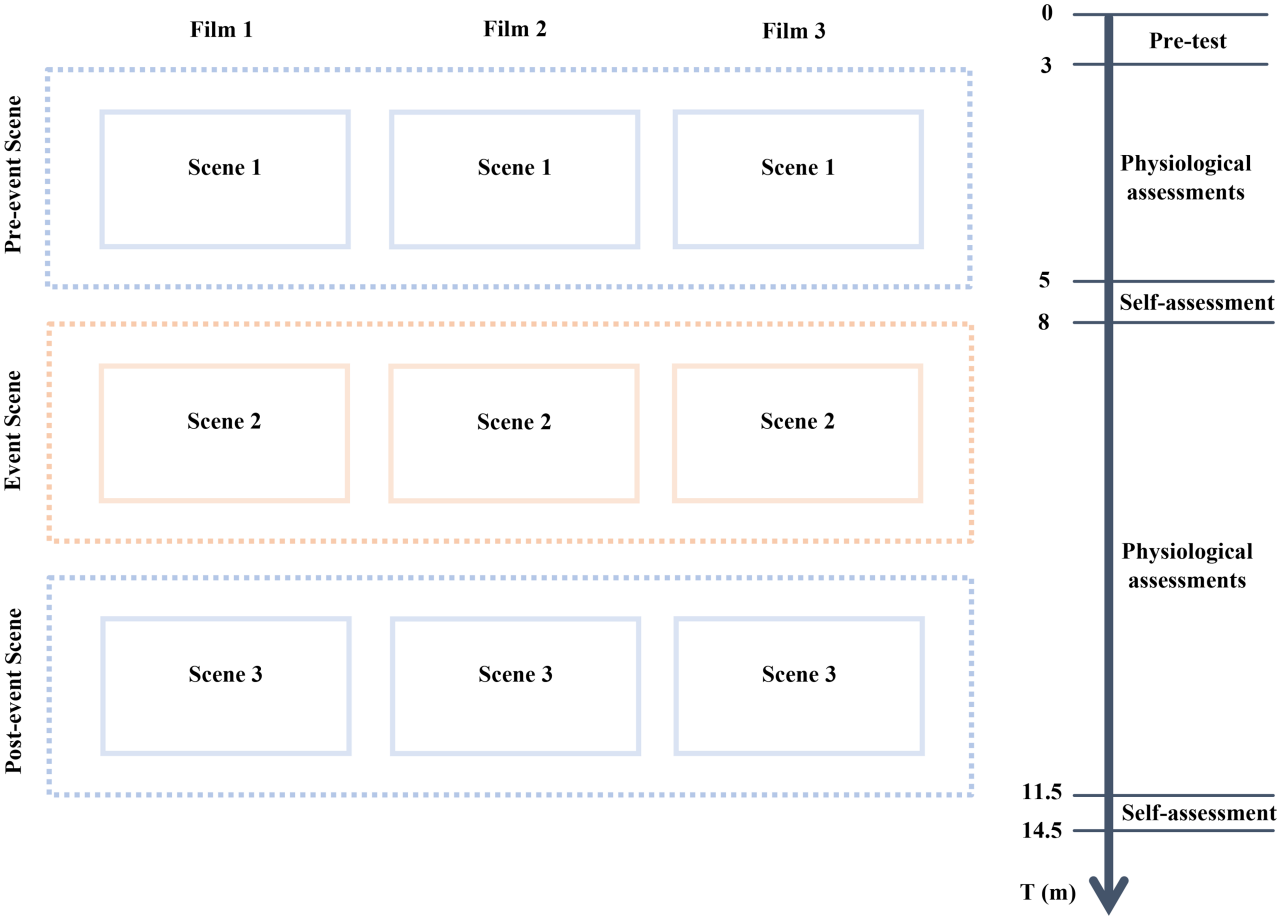
Research design.

The final editions of the film clips were compiled with approximately 5 min each for the experiment. The pre-event and post-event scenes are exactly the same, with a two-minute duration to narrate the everyday life house atmosphere, whereas the middle one-and-a-half-minute event scene shows the evoking event that occurred in the pre-and post-event spaces. We specifically chose the home space to represent the most lived, connected, and private space. Individuals in the pre-event scene familiarize themselves with the architectural features of home spaces, while the middle event scenes show that the same depicted elements shatter and destroy. In the post-event scene, subjects watched the same pre-event scene to help us understand if their previous mnemonic perception of the event scene has affected perceiving the post-event ordinary and banal spaces. Hence, we tested participants’ memory and emotional responses to extract their psychophysiological changes by capturing their biofeedback and psychological questionnaire on film space qualities. These assessments were conducted in three phases: baseline pre-test, during the test, and post-test (Figure 2).

### 2.3. Psychological self-assessment

We collected data using a psychological questionnaire related to space quality appraisal during the film screening in the same test environments between 11 a.m. and 2 p.m. with a constant temperature of 25°C. The NAQ questionnaire (Zawidzki, 2016a; to be completed with pencil and paper) was prepared to assess the geometrical features of architectural spaces in the three films in which the questionnaire items related to the spatial perception of a space. The NAQ test consists of 20 items to assess the quality of the architectural space that includes: *Dynamic/Dull, Attractive/Repelling, Arranged/Chaotic, Natural/Artificial, Interesting/Boring, Comforting/Disturbing, Harmonious/Cacophonous, Functional/Dysfunctional, Social/Unsocial, Cozy/Harsh, Inspiring/Unaspiring, Diverse/Plain, Accessible/Inaccessible, Vital/Fatal, Sensible/Imperceptible, Expressive/Neutral, Emotional/Cool, Memorable/Forgettable, Communicative/Cool,* and *Democratic/Authoritarian.* Each of the two opposite keywords is scored within the range of-3, −2, −1, 0, +1, +2, +3, from which the participants give negative points to negative keywords and positive attributes to positive keywords in each item. The zero scores represent no sense of emotional feelings in each item.

In the pre-test phase of the experiment, we asked participants to sit in a chair for a few minutes to ensure they were relaxed. Then we briefed the experiment procedure and asked them to focus on the architectural characteristics and related changes before and after the events. Once the subjects understood the process and that the test evaluation relates to spatial quality assessments, we started the film screening phase using a 15-inch laptop with a loudspeaker and then paused the first film right after the end of the pre-event scene. At this point, we asked individuals to complete the NAQ questionnaire for the pre-event film. After the self-assessment completion, we showed the event scene and subsequent post-event scene (the exact same scene as the pre-event scene) without pausing to finish the screening of the first movie. At this time, subjects were asked to fill out the NAQ assessment again to evaluate stimuli intensities related to architectural elements. We designed the experiment in this manner since we expected that the events of the middle scene between two similar ordinary scenes would elicit participants’ cognitive load and differentiate between their pre-and post-event NAQ assessment scores. As the NAQ evaluation of the post-event scene was assumed to be affected by the event scene, we repeated the experiment for films two and three by dividing subjects into three groups for each film.

### 2.4. Physiological data collection

During the experiment, the biofeedback indicators of HR, SBP, DBP, BT, and SRL for measuring sweating on the palm surface were recorded for each participant. The HR and SRL indicators were monitored and recorded throughout the film’s screening, while the BT and BP indicators were captured before and after each pre-event and post-event scene. All the biofeedback indicators were also recorded during the pre-test and resting mode as baseline indicators. The physiological data were imported to SPSS software for analysis. The detailed procedure for collecting the biofeedback data conducted in this experiment has been discussed in a previous study (Sakhaei et al., 2022b). Compared to the earlier study, this experiment focuses on the correlation and regression analyses between architectural space qualities and physiological changes in a detailed quantitative approach. In addition, we conducted observational analyses to record any apparent changes in participants’ pupils, skin tone, or feeling chills or goosebumps to validate physiological changes. The analysis of variance (ANOVA) was conducted to compare different variables in the three films. Simple and multiple linear regression was conducted to analyze the impact of variables on space quality. Spearman correlation coefficient analyses were also used to determine the association between variables. *p*-values less than 0.05 were regarded as statistically significant.

## 3. Results

### 3.1. Descriptive statistics of psychophysiological variables

The descriptive statistics of physiological variables of HR, SRL, SBP, DBP, BT, and psychological assessment of NAQ for each film during the pre-test, pre-event, and post-event are reported in Table 2. The ANOVA analyses signified variables’ changes between pre-event and post-event. Accordingly, the pre-and post-event differences of HR (*p*-value < 0.001), SRL (*p*-value < 0.001), SBP (*p*-value = 0.002), DBP (*p*-value = 0.010), BT (*p*-value < 0.001), and NAQ (*p*-value = 0.003) were meaningful in the three films. The NAQ variables of Harsh (−3.00 in Film 1, −3.10 in Film 2, and-3.60 in Film 3), Chaotic (−2.80 in Film 1, −2.80 in Film 2, and-3.10 in Film 3) and Artificial (−1.80 in Film 1, −1.30 in Film 2, and-3.00 in Film 3) depicted the highest negative differences. In contrast, the Diverse variable in NAQ (−0.50 in Film 1, +0.90 in Film 2, and + 0.50 in Film 3) illustrated the highest positive differences compared to other items. The analyzed data from participants’ subjective appraisal demonstrated that the emotional arousal followed by the disruptive events decelerated the spatial quality scores, making participants’ evaluation more homogenized compared to the pre-event evaluation. Spatial stimulus appeared to have decreased the semantic differentiation of the perceived space quality. The overall descriptive statistics report showed that the spatial stimuli in all three films caused a significant change in the physiological variables captured by participants, depicting that the changes in assessing the films’ space qualities before and after the event were meaningful.

**Table 2:** Descriptive statistics of variables by film.

Variables	Film	*N*	Mean ± SD	Median (Q1, Q3)	*p*-Value
Pre-test HR	1	30	80.90 ± 16.59	80.5 (66.0, 92.0)	0.650
	2	30	79.30 ± 13.86	83.5 (71.0, 90.0)	
	3	30	77.50 ± 11.61	82.0 (66.0, 85.0)	
Pre-event HR	1	30	81.83 ± 16.60	82.3 (66.5, 94.0)	0.261
	2	30	78.25 ± 12.59	81.0 (67.3, 88.0)	
	3	30	76.08 ± 11.07	79.5 (63.0, 84.0)	
Post-event HR	1	30	78.80 ± 14.41	81.9 (66.0, 87.8)	0.971
	2	30	79.48 ± 11.20	81.8 (68.3, 88.5)	
	3	30	79.48 ± 11.30	80.9 (69.5, 90.3)	
Difference of HR	1	30	−3.03 ± 3.70	−3.4 (−5.5, 1.0)	**<0.001**
	2	30	1.23 ± 3.19	0.8 (−0.5, 3.0)	
	3	30	3.40 ± 3.87	3.6 (−0.5, 6.5)	
Pre-test SRL	1	30	2361.77 ± 2811.31	1330.0 (570.0, 2560.0)	0.260
	2	30	1334.83 ± 1179.29	965.0 (505.0, 1750.0)	
	3	30	1287.85 ± 1161.79	1070.0 (447.0, 1660.0)	
Pre-event SRL	1	30	2145.42 ± 2315.59	1496.3 (513.8, 2470.0)	0.412
	2	30	1501.50 ± 1347.55	1120.0 (695.0, 1885.0)	
	3	30	1417.62 ± 1347.35	1091.3 (427.8, 1860.0)	
Post-event SRL	1	30	1485.68 ± 1273.91	1088.8 (420.3, 2030.0)	0.971
	2	30	1501.50 ± 1347.55	1120.0 (695.0, 1885.0)	
	3	30	1457.34 ± 1288.78	1182.5 (471.3, 1862.5)	
Difference of SRL	1	30	−659.74 ± 1122.88	−85.5 (−862.5, 15.8)	**<0.001**
	2	30	0.00 ± 0.00	0.0 (0.0, 0.0)	
	3	30	39.73 ± 137.71	48.0 (−2.2, 130.0)	
Pre-test SBP	1	30	120.10 ± 12.04	125.0 (111.0, 129.0)	0.217
	2	30	115.30 ± 12.14	114.5 (102.0, 128.0)	
	3	30	115.70 ± 10.87	117.5 (107.0, 125.0)	
Pre-event SBP	1	30	117.70 ± 9.60	118.0 (108.0, 127.0)	**0.019**
	2	30	109.80 ± 10.11	112.0 (102.0, 118.0)	
	3	30	113.30 ± 11.98	112.5 (103.0, 126.0)	
Post-event SBP	1	30	113.30 ± 12.14	109.5 (107.0, 122.0)	0.707
	2	30	111.90 ± 11.10	112.0 (106.0, 122.0)	
	3	30	110.90 ± 10.28	115.0 (103.0, 117.0)	
Difference of SBP	1	30	−4.40 ± 4.80	−4.5 (−8.0, −1.0)	**0.002**
	2	30	2.10 ± 8.43	3.5 (−3.0, 6.0)	
	3	30	−2.40 ± 7.49	0.0 (−10.0, 5.0)	
Pre-test DBP	1	30	76.80 ± 9.22	75.0 (68.0, 83.0)	**0.015**
	2	30	72.90 ± 4.51	73.0 (68.0, 78.0)	
	3	30	71.90 ± 5.69	72.0 (69.0, 75.0)	
Pre-event DBP	1	30	77.50 ± 6.72	74.5 (72.0, 80.0)	**0.003**
	2	30	74.00 ± 7.64	73.0 (70.0, 79.0)	
	3	30	71.40 ± 5.50	72.0 (67.0, 76.0)	
Post-event DBP	1	30	75.80 ± 6.79	76.0 (71.0, 79.0)	0.512
	2	30	73.50 ± 8.09	73.0 (69.0, 80.0)	
	3	30	73.80 ± 9.85	73.0 (68.0, 81.0)	
Difference of DBP	1	30	−1.70 ± 4.60	−2.5 (−5.0, 0.0)	**0.010**
	2	30	−0.50 ± 2.85	1.0 (−4.0, 2.0)	
	3	30	2.40 ± 7.32	2.5 (1.0, 4.0)	
Pre-test BT	1	30	35.57 ± 0.36	35.6 (35.3, 35.9)	**0.020**
	2	30	35.75 ± 0.28	35.8 (35.6, 35.9)	
	3	30	35.76 ± 0.20	35.8 (35.6, 35.9)	
Pre-event BT	1	30	35.66 ± 0.34	35.6 (35.5, 35.9)	0.187
	2	30	35.76 ± 0.26	35.8 (35.6, 36.0)	
	3	30	35.79 ± 0.25	35.9 (35.5, 36.0)	
Post-event BT	1	30	35.88 ± 0.29	35.9 (35.7, 36.1)	0.115
	2	30	35.82 ± 0.24	35.9 (35.7, 36.0)	
	3	30	35.73 ± 0.29	35.7 (35.5, 36.0)	
Difference of BT	1	30	0.22 ± 0.20	0.3 (0.0, 0.3)	**<0.001**
	2	30	0.06 ± 0.18	0.1 (−0.1, 0.2)	
	3	30	−0.06 ± 0.19	0.0 (−0.2, 0.1)	
Pre-event NAQ	1	30	5.01 ± 0.47	5.0 (4.8, 5.4)	**<0.001**
	2	30	5.06 ± 0.67	5.1 (4.6, 5.5)	
	3	30	3.75 ± 1.00	4.0 (2.8, 4.3)	
Post-event NAQ	1	30	4.38 ± 0.88	4.5 (3.7, 4.9)	**0.026**
	2	30	4.40 ± 0.68	4.4 (3.9, 5.0)	
	3	30	3.92 ± 0.72	4.0 (3.4, 4.6)	
Difference of NAQ	1	30	−0.63 ± 1.13	−0.5 (−1.5, −0.3)	**0.003**
	2	30	−0.66 ± 0.95	−0.6 (−0.9, 0.0)	
	3	30	0.17 ± 0.95	0.5 (−0.5, 0.8)	

The analysis of variance (ANOVA) was used to compare the mean of normal variables between different films. The Kruskal–Wallis test was used to compare the median of non-normal variables between different films. Heart rate (HR), skin resistance level (SRL), systolic blood pressure (SBP), diastolic blood pressure (DBP), body temperature (BT), normalized accumulated quality (NAQ). Bold values mean that they are significant.

### 3.2. Correlation analyses

**Figure 3:**
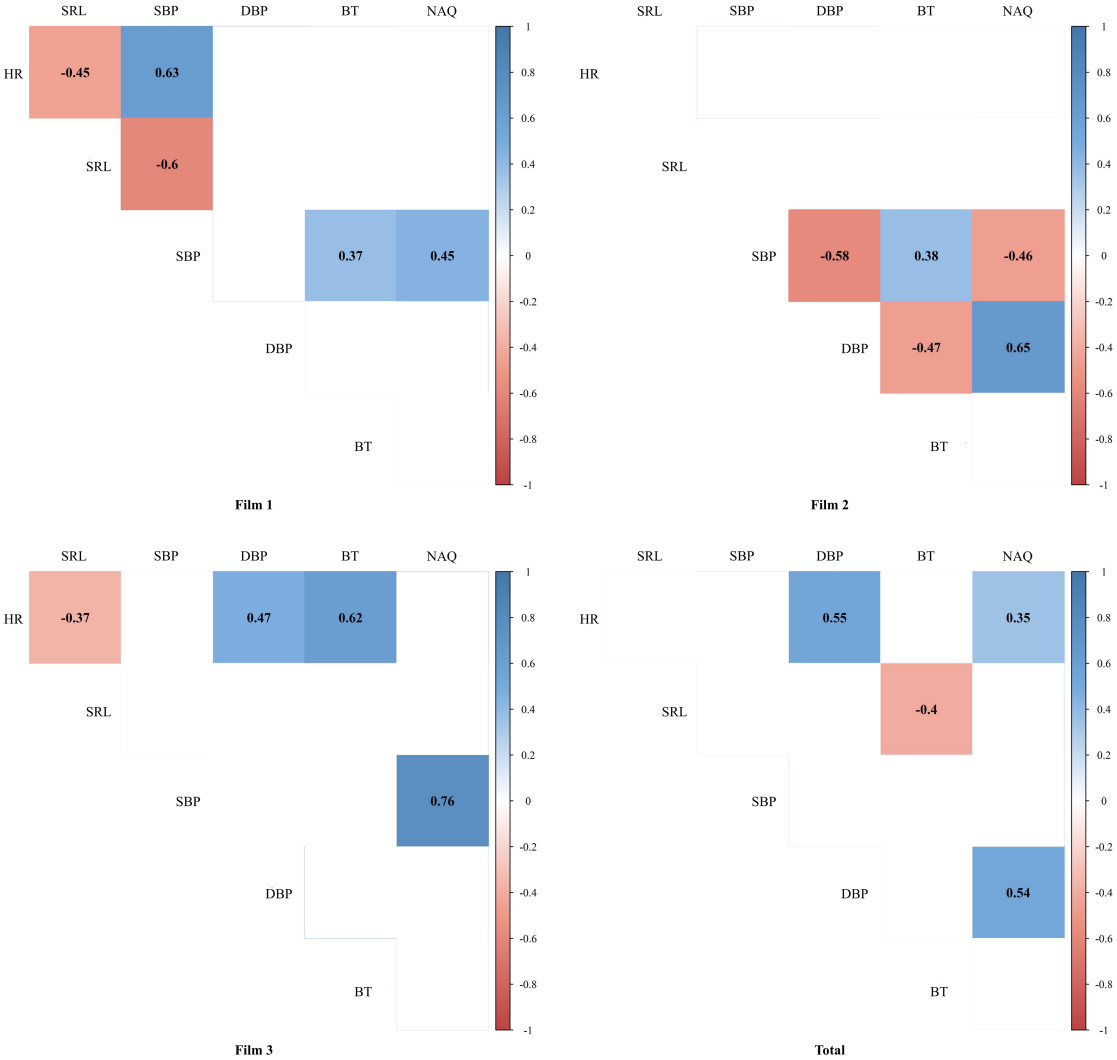
The Spearman correlation coefficient between variable differences.

Figure 3 shows the matrixes of associations between variables in total and for each of the three films separately. In Film 1, NAQ was significantly associated with SBP (0.45; 95% CI: 0.10, 0.70, *p*-value = 0.013). The matrix also showed a meaningful association between HR and SRL (−0.46; 95% CI: −0.71, −0.10, *p*-value = 0.012), HR and SBP (0.63; 95% CI: 0.33, 0.81, *p*-value < 0.001), SRL and SBP (−0.60; 95% CI: −0.79, −0.29, *p*-value = 0.001), as well as SBP and BT (0.37; 95% CI: 0.00, 0.65, *p*-value = 0.042). In Film 2, NAQ was also meaningfully associated with SBP (−0.46; 95% CI: −0.71, −0.11, *p*-value = 0.010) and DBP (0.65; 95% CI: 0.37, 0.82, *p*-value < 0.001). Additionally, significant associations between BT and SBP (−0.38; 95% CI: 0.01, 0.66, *p*-value = 0.040), BT and DBP (−0.47; 95% CI: −0.71, −0.12, *p*-value = 0.010), as well as DBP and SBP (−0.58; 95% CI: −0.78, −0.27, *p*-value = 0.001) was observed. In Film 3, NAQ was associated only with SBP factor (0.76; 95% CI: 0.54, 0.88, *p*-value < 0.001). Besides, HR was associated with the three factors of BT (0.62; 95% CI: 0.32, 0.81, *p*-value < 0.001), DBP (0.47; 95% CI: 0.13, 0.72, *p*-value = 0.008), and SRL (−0.37; 95% CI: −0.65, 0.00, *p*-value = 0.044). In the average of the three films in total, NAQ was associated with HR (0.35; 95% CI: 0.15, 0.53, *p*-value = 0.001) and DBP (0.54; 95% CI: 0.36, 0.67, *p*-value < 0.001), illustrating that there was a meaningful relationship between understanding the space quality and physiological changes with the presence of emotion-eliciting stimuli in space. Also, there were significant associations between BT and SRL (−0.40; 95% CI: −0.57, −0.21, *p*-value < 0.001), and DBP and HR (0.55; 95% CI: 0.38, 0.68, *p*-value < 0.001). Overall, the matrixes of associations between variables demonstrated a meaningful association between the NAQ factor and physiological changes of HR, SBP, and DBP factors derived from individuals. The Supplementary Table S1 shows the detailed spearman correlation coefficient (95% CI) between variables for the three films.

### 3.3. Regression analyses

The adjusted and unadjusted impact of variable differences on the NAQ difference is reported in Table 3. Based on the linear regression results, a meaningful impact was observed on NAQ for unadjusted HR in Film 3 (0.09; 95% CI: 0.00, 0.18, *p*-value = 0.045) and in total of the three films (0.06; 95% CI: 0.01, 0.11, *p*-value = 0.020). Regarding the unadjusted impact of SBP on NAQ, a significant impact was observed in Film 1 (0.09; 95% CI: 0.00, 0.17, *p*-value < 0.001), Film 2 (−0.07; 95% CI: −0.10, −0.04, *p*-value < 0.001), and Film 3 (0.09; 95% CI: 0.05, 0.12, *p*-value < 0.001). As for the adjusted impact of SBP on NAQ, a meaningful impact was also reported for Film 1 (0.10; 95% CI: 0.03, 0.16, *p*-value = 0.008), Film 2 (−0.05; 95% CI: −0.09, −0.01, *p*-value = 0.011), and Film 3 (0.10; 95% CI: 0.07, 0.12, *p*-value < 0.001). According to the unadjusted impact of DBP on NAQ, a significant impact was observed in Film 1 (0.13; 95% CI: 0.05, 0.21, *p*-value = 0.002), Film 2 (0.17; 95% CI: 0.06, 0.28, *p*-value = 0.003), Film 3 (0.06; 95% CI: 0.02, 0.11, *p*-value = 0.009), and total of the three films (0.10; 95% CI: 0.07, 0.14, *p*-value < 0.001). A significant adjusted impact of DBP on NAQ was found in Film 1 (0.14; 95% CI: 0.07, 0.21, *p*-value < 0.001), Film 3 (0.07; 95% CI: 0.04, 0.10, *p*-value < 0.001), and total of the three films (0.11; 95% CI: 0.06, 0.15, *p*-value < 0.001). Based on the results derived from the simple and multiple linear regressions, the physiological factors on HR and BP significantly impacted perceiving the quality of space. The reports showed that the emotional changes of participants after observing spatial stimuli significantly impacted the subjective assessment of space.

**Table 3:** The unadjusted and adjusted impact of variable differences on the NAQ difference by film.

Variables	Films	Unadjusted		Adjusted	
		*B* (95% CI)	*p*-value	*B* (95% CI)	*p*-value
Difference of HR	1	0.05 (−0.07, 0.16)	0.429		
	2	−0.07 (−0.18, 0.04)	0.207		
	**3**	**0.09 (0.00, 0.18)**	**0.045**	0.02 (−0.04, 0.07)	0.507
	**Total**	**0.06 (0.01, 0.11)**	**0.020**	−0.01 (−0.06, 0.04)	0.779
Difference of SRL	1	0.00 (0.00, 0.00)	0.424		
	2	0.00 (0.00, 0.00)	0.478		
	3				
	Total	0.00 (0.00, 0.00)	0.143		
Difference of SBP	**1**	**0.09 (0.00, 0.17)**	**0.050**	**0.10 (0.03, 0.16)**	**0.008**
	**2**	**−0.07 (−0.10, −0.04)**	**<0.001**	**−0.05 (−0.09, −0.01)**	**0.011**
	**3**	**0.09 (0.05, 0.12)**	**<0.001**	**0.10 (0.07, 0.12)**	**<0.001**
	Total	0.01 (−0.02, 0.04)	0.647		
Difference of DBP	**1**	**0.13 (0.05, 0.21)**	**0.002**	**0.14 (0.07, 0.21)**	**<0.001**
	**2**	**0.17 (0.06, 0.28)**	**0.003**	0.09 (−0.03, 0.21)	0.138
	**3**	**0.06 (0.02, 0.11)**	**0.009**	**0.07 (0.04, 0.10)**	**<0.001**
	**Total**	**0.10 (0.07, 0.14)**	**<0.001**	**0.11 (0.06, 0.15)**	**<0.001**
Difference of BT	1	0.61 (−1.55, 2.76)	0.567		
	2	−0.34 (−2.35, 1.67)	0.734		
	3	1.06 (−0.86, 2.97)	0.267		
	Total	−0.38 (−1.41, 0.64)	0.459		

Simple and multiple linear regression was used to evaluate the impact of variables on the NAQ difference

Heart rate (HR), skin resistance level (SRL), systolic blood pressure (SBP), diastolic blood pressure (DBP), body temperature (BT), normalized accumulated quality (NAQ). Bold values mean that they are significant.

## 4. Discussion

The present study tried to establish the relationship between physiological changes and perceived environmental quality in space derived from Zawidzki’s (2016a) Human Subjective Evaluation (HSE) of space. Since Zawidzki’s model presented the 20 items to define normalized space quality factors, we adopted and implemented spatial stimuli in our test model and captured the differences between pre-and post-event scenes to find physiological parameters’ impacts on space cognition. The screened affect-based video clips representing architectural spaces were divided into three phases of pre-event, event, and post-event to controllably capture the emotional changes in participants, thus letting us reveal the impact of physiological variables on normalized space quality factors. The descriptive statistics of variables from the ANOVA analyses highlighted significant transformations in physiological and psychological states. According to our results, the NAQ test’s negative factors of Chaotic, Harsh and Artificial substantially impacted post-event evaluations, while the Diverse factor was the only positive item to be influenced by the stimuli in the film spaces. Based on this observation, we may argue that the spatial features of being Chaotic, Harsh, Artificial and Diverse can noticeably affect physiological changes of our participants.

Our experimental model, which relied on experiencing movement in space and activation of attention, validated the affective role of auditory and visual contents in evaluating human behavior (Torresin et al., 2018; Schweiker et al., 2020). Based on observational analyses, the extreme concentration of participants during the event scenes can be a sign of their deep focus condition and distraction from spatial stimuli, as earlier discussed in the literature (Wais et al., 2010; Buetti and Lleras, 2016). As previously explored (Elliott and Quintino, 2019), these changes can be a sign of autonomic bodily responses to stimuli, which were changes in architectural forms in this experiment. The meaningful changes in physiological feedback in subjects illustrated that emotional arousal may have played an influential role when participants were observing different scenes, as echoed in multiple studies (Altman and Stodols, 1987; Cohen and Areni, 1991; Cacioppo et al., 2000; Radberg and Steffner, 2003; Cho and Kim, 2017). Also, the NAQ’s significant changes between pre-event and post-event assessments validated the previous claim that subjects’ emotional and mental states can be altered when being exposed to various spatial stimuli (Persiani et al., 2021).

In this study, we employed the unexpected and deconstructive event scenes as sensory stimuli in space to elicit emotional behavior and induce a bottom-up cognitive process (Kayser and Logothetis, 2007). The exploration of architectural characteristics and related evaluation differences between normal and stimulus-affected scenes highlighted a meaningful positive correlation between space quality perception and physiological factors of HR and BP. This correlation validated previous studies that visual stimuli may be a practical context to perceive the surrounding environment and architectural space appraisals (Godfroy-Cooper et al., 2015; Schreuder et al., 2016). It also explained the Chatterjee model in which the affective-emotional output in space can evoke attention to notice architectural features (Coburn et al., 2017).

The regression results established a strong impact of the physiological factor of HR and BP (both SBP and DBP) on emotion-triggered stimuli perception and subsequent environmental quality appraisals. Our analyses of the adjusted and unadjusted impact of physiological variables on NAQ may possibly explain the James-Lange theory (James, 1884), Cannon-Bard theory (Cannon, 1927), and Schachter-Singer theory (Schachter and Singer, 1962). Besides, observational techniques from the participants’ apparent facial expressions revealed significant skin tone and pupil changes from pre-event to event scenes, validating the Facial Feedback theory (Lanzetta et al., 1976) mentioned earlier. During the event scenes, individuals witnessed noticeable alterations in color, light, sound, and texture of architectural elements, while their physiological states and arousal level seemed to have changed meaningfully. Based on relevant literature (Lang, 1988; Nasar, 1997; Sanoff, 2016; Cho and Kim, 2017), we can validate that the sensory stimuli activated subjects’ ANS system and, therefore, caused HR, BP, BT, and SRL differences. Although significant correlation and impact between NAQ and HR and BP were observed, no meaningful association or impact was reported for NAQ with BT and SRL.

According to the reports from the descriptive analyses of variables, simulating movement-based exploration of stimulus-affected spaces created a sense of integrated motion and emotion and validated Bruno’s claim about the close relationship between sight and site, motion and emotion in architecture (Bruno, 2002). The cinematic context in this test could possibly connect the psychological states bonded to the built environment with emotional feedback that appeared in physiological changes. NAQ factors of Harsh, Chaotic, Artificial, and Diverse were the most noticeable items to affect psychological assessments of space qualities. We can argue that participants’ evaluation of space showed significant attention to architectural details as they evaluated the post-event space as more diverse, artificial, or chaotic. These items can explain the significant association of NAQ with BT and BP and also the meaningful impact of physiological variables’ changes on NAQ differences.

The results derived from the experiment validated the association between architectural spaces’ psychophysiological responses and environmental characteristics’ appraisal, as previously discussed by Franz and colleagues (Franz et al., 2005). We also demonstrated that film contents depicting built environments could act as a proper medium to elicit emotional changes and subsequent physiological signals in individuals, validating Wu and colleagues in a similar study (Wu et al., 2008). We chose Zawidzki’s normalized accumulated quality of space from subjective assessments of people, and achieved a significant difference in viewers’ responses due to the successful implementation of unexpected stimuli. This alteration between normalized and event-based space appraisal helped us capture three human physiological indicators’ association with space qualities like Harsh, Chaotic, Artificial, and Diverse.

## 5. Conclusion

In this study, we tried to illustrate the interrelationships between architectural space perception and physiological attributes to environmental characteristics through the mediation of cinematic context. The result of this experiment shows that during the space quality judgments, physiological changes caused by emotional arousal could possibly be associated with the psychological assessments. Using stimuli-affected video content to depict architectural spaces appeared to be efficient in extracting the impact of physiological states and human cognitive responses on space quality. Implementing disruptive events related to architectural characteristics can be practicable to improve the cognitive load to perceive and appraise the surrounding built environment. This research also demonstrated that noticing physiological characteristics during the space appraisal may help understand the human-environment interaction in a multi-layered approach by considering architectural design, spatial cognition, and emotional arousal in an integrated manner.

There are a number of limitations in the current study. Since the items in NAQ variables were limited, and there are many other space characteristics to explore, future experiments can involve different space appraisal surveys to find their relationship with human behavior. In this project, to control the feasibility and validity of film scenes, we chose our scene among three films, while the diversity of film scenes and film numbers can be extended to illustrate more architectural space characteristics. Using Virtual Reality techniques in future research can also bring a more accurate psychophysiological analysis of spaces by modifying the architectural elements and movements. Regarding physiological feedback as one of the critical indicators of human behavior and bodily response to environmental stimuli, more advanced tools like eye-tracking technology, respiratory measurements, and neuro-imaging techniques can be performed in the future to reach a transcendent understanding between physiology and spatial cognition. In addition, some physiological responses from emotional arousal, like HR, can be biased as it may elevate or decelerate among participants during emotional arousal. Hence, future discussions can focus on optimizing the experiments to justify controversial data during exposure to unexpected stimuli. Apart from this, exploring the relationship between physiological alterations and space structures in a larger statistical population can achieve more diverse data collection to explore physiological response differences within different nationalities, thus performing a comparative analysis. Such improvements in research data and experimental data retrieval can advance the role of architectural space features, as well as enhance quantitative research in spatial cognition.

## Data availability statement

The original contributions presented in the study are included in the article/Supplementary material, further inquiries can be directed to the corresponding author.

## Ethics statement

The studies involving human participants were reviewed and approved by Department of Physiology, Tarbiat Modares University. Written informed consent for participation was not required for this study in accordance with the national legislation and the institutional requirements.

## Author contributions

HS: conceptualization, methodology, writing original draft, funding acquisition, and investigation. HS and MAL: data curation, software, visualization and formal analysis. HS and NG: review, editing, validation, project administration and supervision.

## Conflict of interest

The authors declare that the research was conducted in the absence of any commercial or financial relationships that could be construed as a potential conflict of interest.

## Publisher’s note

All claims expressed in this article are solely those of the authors and do not necessarily represent those of their affiliated organizations, or those of the publisher, the editors and the reviewers. Any product that may be evaluated in this article, or claim that may be made by its manufacturer, is not guaranteed or endorsed by the publisher.

## Supplementary material

The Supplementary material for this article can befound online at: https://www.frontiersin.org/articles/10.3389/fpsyg.2022.1012758/full#supplementary-material

SUPPLEMENTARY TABLE 1 The Spearman correlation coefficient (95% CI) between variables.
