# Supplementary material for: Assessing the association between subjective evaluation of space qualities and physiological responses through cinematic environments’ emotion-eliciting stimuli
Source: Front Psychol. 2023 Jan 12;13:1012758. doi: 10.3389/fpsyg.2022.1012758 (PMC9879063; doi:10.3389/fpsyg.2022.1012758)
Supplement: SUPPLEMENTARY TABLE 1 — The Spearman correlation coefficient (95% CI) between variables. [file Table_1.docx]

**Table S1.** The Spearman correlation coefficient (95% CI) between variables.

| **Films** | **Variables** | **r (95% CI)^a,b^** | **P-value** |
| --- | --- | --- | --- |
| Total | Difference of HR - Difference of SRL | 0.01 (-0.20, 0.23) | 0.893 |
|  | Difference of HR - Difference of SBP | 0.17 (-0.05, 0.37) | 0.121 |
|  | **Difference of HR - Difference of DBP** | **0.55 (0.38, 0.68)** | **<0.001** |
|  | Difference of HR - Difference of BT | -0.01 (-0.23, 0.20) | 0.902 |
|  | **Difference of HR - Difference of NAQ** | **0.35 (0.15, 0.53)** | **0.001** |
|  | Difference of SRL - Difference of SBP | 0.06 (-0.15, 0.27) | 0.562 |
|  | Difference of SRL - Difference of DBP | 0.12 (-0.10, 0.32) | 0.280 |
|  | **Difference of SRL** **- Difference of BT** | **-0.40 (-0.57, -0.21)** | **<0.001** |
|  | Difference of SRL - Difference of NAQ | 0.18 (-0.04, 0.38) | 0.092 |
|  | Difference of SBP - Difference of DBP | -0.09 (-0.30, 0.12) | 0.386 |
|  | Difference of SBP - Difference of BT | 0.11 (-0.11, 0.31) | 0.313 |
|  | Difference of SBP - Difference of NAQ | 0.17 (-0.05, 0.37) | 0.119 |
|  | Difference of DBP - Difference of BT | -0.03 (-0.24, 0.19) | 0.795 |
|  | **Difference of DBP - Difference of NAQ** | **0.54 (0.36, 0.67)** | **<0.001** |
|  | Difference of BT - Difference of NAQ | -0.05 (-0.26, 0.16) | 0.624 |
| 1 | **Difference of HR - Difference of SRL** | **-0.46 (-0.71, -0.10)** | **0.012** |
|  | **Difference of HR - Difference of SBP** | **0.63 (0.33, 0.81)** | **<0.001** |
|  | Difference of HR - Difference of DBP | 0.35 (-0.03, 0.64) | 0.061 |
|  | Difference of HR - Difference of BT | 0.25 (-0.14, 0.57) | 0.190 |
|  | Difference of HR - Difference of NAQ | 0.12 (-0.27, 0.47) | 0.540 |
|  | **Difference of SRL** **- Difference of SBP** | **-0.60 (-0.79, -0.29)** | **0.001** |
|  | Difference of SRL - Difference of DBP | 0.33 (-0.04, 0.63) | 0.071 |
|  | Difference of SRL - Difference of BT | -0.23 (-0.56, 0.15) | 0.214 |
|  | Difference of SRL - Difference of NAQ | 0.07 (-0.31, 0.43) | 0.723 |
|  | Difference of SBP - Difference of DBP | -0.20 (-0.53, 0.19) | 0.294 |
|  | **Difference of SBP - Difference of BT** | **0.37 (0.00, 0.65)** | **0.042** |
|  | **Difference of SBP - Difference of NAQ** | **0.45 (0.10, 0.70)** | **0.013** |
|  | Difference of DBP - Difference of BT | 0.05 (-0.33, 0.41) | 0.796 |
|  | Difference of DBP - Difference of NAQ | 0.22 (-0.16, 0.55) | 0.239 |
|  | Difference of BT - Difference of NAQ | -0.03 (-0.40, 0.34) | 0.870 |
| 2 | Difference of HR - Difference of SRL | ----- | ----- |
|  | Difference of HR - Difference of SBP | -0.15 (-0.49, 0.23) | 0.424 |
|  | Difference of HR - Difference of DBP | 0.14 (-0.24, 0.49) | 0.454 |
|  | Difference of HR - Difference of BT | 0.04 (-0.34, 0.40) | 0.846 |
|  | Difference of HR - Difference of NAQ | -0.06 (-0.42, 0.32) | 0.750 |
|  | Difference of SRL - Difference of SBP | ----- | ----- |
|  | Difference of SRL - Difference of DBP | ----- | ----- |
|  | Difference of SRL - Difference of BT | ----- | ----- |
|  | Difference of SRL - Difference of NAQ | ----- | ----- |
|  | **Difference of SBP - Difference of DBP** | **-0.58 (-0.78, -0.27)** | **0.001** |
|  | **Difference of SBP - Difference of BT** | **0.38 (0.01, 0.66)** | **0.040** |
|  | **Difference of SBP - Difference of NAQ** | **-0.46 (-0.71, -0.11)** | **0.010** |
|  | **Difference of DBP - Difference of BT** | **-0.47 (-0.71, -0.12)** | **0.010** |
|  | **Difference of DBP - Difference of NAQ** | **0.65 (0.37, 0.82)** | **<0.001** |
|  | Difference of BT - Difference of NAQ | -0.04 (-0.41, 0.33) | 0.820 |
| 3 | **Difference of HR - Difference of SRL** | **-0.37 (-0.65, 0.00)** | **0.044** |
|  | Difference of HR - Difference of SBP | 0.03 (-0.34, 0.40) | 0.870 |
|  | **Difference of HR - Difference of DBP** | **0.47 (0.13, 0.72)** | **0.008** |
|  | **Difference of HR - Difference of BT** | **0.62 (0.32, 0.81)** | **<0.001** |
|  | Difference of HR - Difference of NAQ | 0.30 (-0.08, 0.60) | 0.111 |
|  | Difference of SRL - Difference of SBP | -0.01 (-0.38, 0.36) | 0.948 |
|  | Difference of SRL - Difference of DBP | -0.09 (-0.44, 0.29) | 0.651 |
|  | Difference of SRL - Difference of BT | -0.33 (-0.62, 0.05) | 0.080 |
|  | Difference of SRL - Difference of NAQ | -0.20 (-0.53, 0.18) | 0.289 |
|  | Difference of SBP - Difference of DBP | 0.00 (-0.37, 0.37) | 1.000 |
|  | Difference of SBP - Difference of BT | 0.11 (-0.27, 0.46) | 0.573 |
|  | **Difference of SBP - Difference of NAQ** | **0.76 (0.54, 0.88)** | **<0.001** |
|  | Difference of DBP - Difference of BT | 0.36 (-0.02, 0.64) | 0.054 |
|  | Difference of DBP - Difference of NAQ | 0.16 (-0.22, 0.50) | 0.398 |
|  | Difference of BT - Difference of NAQ | 0.12 (-0.27, 0.47) | 0.540 |

^a^ Estimation is based on Fisher's r-to-z transformation.

^b^ Estimation of the standard error is based on the formula proposed by Fieller, Hartley, and Pearson.
